# Quantum dot conjugated nanobodies for multiplex imaging of protein dynamics at synapses†
†Electronic supplementary information (ESI) available: Detailed materials and methods, supplementary figures, and supporting information videos. See DOI: 10.1039/c7nr09130c


**DOI:** 10.1039/c7nr09130c

**Published:** 2018-05-17

**Authors:** Souvik Modi, Nathalie F. Higgs, David Sheehan, Lewis D. Griffin, Josef T. Kittler

**Affiliations:** a Neuroscience , Physiology and Pharmacology , University College London. Gower Street , London , WC1E 6BT , UK . Email: j.kittler@ucl.ac.uk; b Tata Institute of Fundamental Research , Homi Bhabha Road , Mumbai , 400005 , India; c Department of Computer Science , University College London , UK

## Abstract

Neurons communicate with each other through synapses, which show enrichment for specialized receptors. Although many studies have explored spatial enrichment and diffusion of these receptors in dissociated neurons using single particle tracking, much less is known about their dynamic properties at synapses in complex tissue like brain slices. Here we report the use of smaller and highly specific quantum dots conjugated with a recombinant single domain antibody fragment (V_H_H fragment) against green fluorescent protein to provide information on diffusion of adhesion molecules at the growth cone and neurotransmitter receptors at synapses. Our data reveals that QD-nanobodies can measure neurotransmitter receptor dynamics at both excitatory and inhibitory synapses in primary neuronal cultures as well as in *ex vivo* rat brain slices. We also demonstrate that this approach can be applied to tagging multiple proteins to simultaneously monitor their behavior. Thus, we provide a strategy for multiplex imaging of tagged membrane proteins to study their clustering, diffusion and transport both *in vitro* as well as in native tissue environments such as brain slices.

## Abstract 2

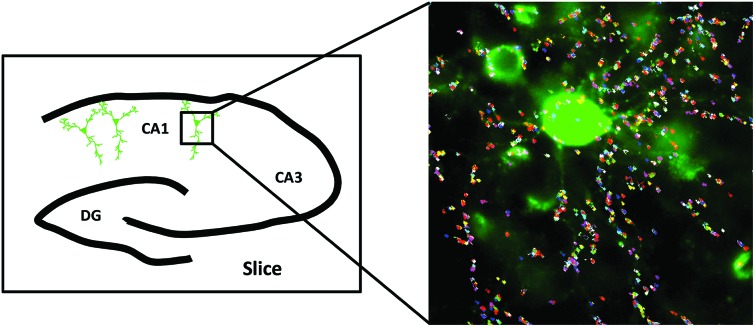
An anti-GFP nanobody conjugated QD optical probe was applied to study single particle tracking *in vitro* and *ex vivo*. This small, highly specific probe recognized GFP when expressed at the cell membrane and reported diffusion dynamics of the underlying target protein.

## Introduction

The cell membrane is described as a ‘fluid mosaic’ environment where specific proteins segregate into microdomains to facilitate downstream signalling.^1^ These microdomains, enriched in lipids, sterols, signalling receptors, transporters, and ion channels are very dynamic and undergo continuous assembly and disassembly due to lateral diffusion in the cell membrane.^1^,^2^ Lateral diffusion of plasma membrane proteins has been studied using optical imaging methods by targeting their extracellular domains with fluorescent markers or ligands, or antibodies conjugated to fluorescent tags, or by using fluorescent protein chimeras.^3^ Ensemble information about fluorescently tagged membrane proteins and their cellular trafficking has been investigated using fluorescent recovery after photobleaching (FRAP), while single-molecule detection methods have provided insight about diffusion properties of individual proteins.^3^,^4^ Single particle tracking (SPT) can follow the fate of individual molecules tagged with antibodies or ligands conjugated to latex beads, organic dyes, nanometer sized colloidal gold nanoparticles or semiconductor nanocrystals (quantum dots; QDs).^5^–^7^ QDs have exceptional brightness, high photostability and can be readily conjugated to biomolecules.^8^ QD conjugated antibodies have been widely used for the direct measurement of the diffusion coefficient of proteins at the plasma membrane and transport of organelles in the cytoplasm.^9^,^10^ Over the years, studies of QD-labeled proteins/receptors, including Epidermal growth factor receptor, potassium channels, CFTR channels or adhesion proteins like integrins or band 3 proteins have revealed active participation of the cytoskeleton for their dynamics.^9^–^14^

Lateral diffusion and clustering of neurotransmitter receptors and ion channels plays a key role in signaling in the central nervous system, where one determinant of synaptic transmission and plasticity is the number of synaptic receptors and their mobility.^15^ Studies following the diffusion and synaptic stability of neurotransmitter receptors, including AMPA, GABA, NMDA, glycine, cannabinoid and acetylcholine receptors have revealed key information about their mobility and altered dynamics during synaptic communication and plasticity.^15^–^20^ Conventional QD labeling using primary and secondary antibodies has been extensively used for tracking organelles, motor proteins, membrane proteins or neuronal receptors. However this approach can result in probe sizes bigger than 40 nm which may restrict access to confined areas such as synapses and subsequently impact on diffusion dynamics of the tagged receptors.^21^,22a Due to their extensive use for tracking protein dynamics there has been a strong impetus to develop improved QD conjugates that can reduce probe sizes.22b,c,d

Here we report the application of QD-nanobodies for studying receptor diffusion at excitatory and inhibitory synapses in dissociated cultures and brain slices. We conjugated QDs to small, high affinity single-domain antibodies (V_H_H only or sdAb) that recognize GFP or RFP.^23^,^24^ These QD-nanobody conjugates can be monitored inside and outside synapses for long time periods using simple widefield microscopy. Our report shows that these QD-nanobody conjugates can be used to probe different aspects of membrane protein dynamics either during development of axons or at established excitatory or inhibitory synapses. Further, we demonstrate that the QD-nanobodies can be used to study GABA_A_ receptor mobility in *ex vivo* brain slices, which increases possibilities of imaging of synaptic receptors in intact tissues with high precision. Finally, we also showed that, these QD-nanobody conjugates can be used to simultaneously monitor multiple proteins expressed in the same cell.

## Experimental section

### Constructs

The N-terminally tagged α2-SEP DNA was a kind gift from S. Moss (Tufts University, Cambridge, MA) and has been described previously.^18^ GluA2-SEP construct was developed by the Malinow lab (University of California, San Diego) and obtained from Addgene (Plasmid #24001).25*a* GPI-RFP was obtained from the Heisenberg lab (Institute of Science and Technology Austria)25*b* GFP-NrCAM and AnkyrinG-GFP constructs were obtained from Dargent Lab (Aix Marseille Université, Marseille).^26^

### Cell culture and transfections

Rat hippocampal neurons were prepared and cultured from embryonic day 18 rat brains for growth cone analysis. Cells were transfected by Amaxa nucleofection as previously described.^18^,25c For transfection of AMPA receptor constructs, lipofectamine 2000 (Life technologies) was used following manufacturers recommendations. Organotypic slices were prepared from postnatal day 7–10 rats and transfected biolistically using a Gene gun (Biorad). For details, see ESI materials and methods.†

### Live cell imaging

Imaging was performed in a widefield microscope with 60× objective coupled to an Andor CCD camera with samples maintained under heated perfusion (4 ml min^–1^ and 35–37 °C) with imaging media (125 mM NaCl, 5 mM KCl, 1 mM MgCl_2_, 2 mM CaCl_2_, 10 mM d-glucose, and 10 mM HEPES and was adjusted to pH 7.4 with NaOH before use). For imaging Organotypic slices, HEPES was replaced with bicarbonate and buffer (126 mM NaCl, 5 mM KCl, 1 mM MgCl_2_, 2 mM CaCl_2_, 10 mM d-glucose, 1 mM Na_2_HPO_4_ and 24 mM NaHCO_3_) was continuously bubbled with 95% CO_2_/5% O_2_ during the whole imaging session.25*d*

### QD detection, tracking and image analysis

Transfected neurons were labeled with QD-nanobody conjugates at room temperature in imaging media containing 10% Horse serum and 1% BSA unless specified. Detection, tracking and analysis of QDs for MSD and diffusion coefficients were performed as described previously.^18^ For details, see ESI materials and methods.† QD tracks for the representation in the figures were constructed using Mosaic particle tracker 2D/3D and stitching was performed using MosaicJ plugin available in ImageJ.25e,f

### Statistical analysis

All experiments were performed on neurons from at least three individual preparations. Unless otherwise stated, *P* values given are from Mann–Whitney tests and values are given as mean ± IQR (25–75%).

## Results and discussion

**Fig. 1:**
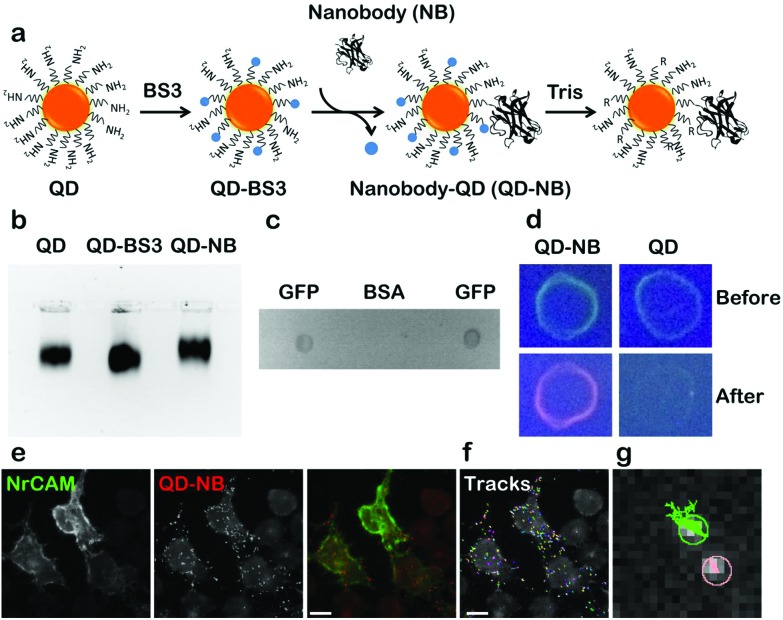
Coupling and characterization of QD-nanobody conjugate. (a) Schematic of QD-nanobody conjugate coupling of 605 nm amino modified QDs to V_H_H only anti GFP nanobody using bifunctional cross linker BS3. (b) Agarose gel electrophoresis of QD conjugates. Free QD and QD conjugates were separated in 2% Agarose-TAE gel and visualized using QD fluorescence after exciting with UV wavelength. QD-BS3 was used as a control to compare gel mobility difference between free QD and QD conjugate. (c) Specificity of QD conjugate was determined using dot blot. GFP and BSA (1 mg ml^–1^) were blotted onto a nitrocellulose membrane, incubated with QD-nanobody conjugates and visualized by placing it under UV. (d) A dot blot as performed in (c) with free QD as a control. Membrane with GFP was incubated with QD conjugate and free QDs. Images were acquired before incubation with QDs (before) and after overnight incubation with QDs (after) at 4 °C. (e–g) Specificity of QD-nanobody conjugates toward GFP-NrCAM expressing HeLa cells. Cells were transfected with GFP-NrCAM and 24 h post-transfection, labelled with QD-nanobody conjugate and imaged in a widefield microscope. Scale bar: 40 μm. QD-NB: QD conjugated nanobodies.

Coupling of QD-nanobody was performed in a two-step process. First amine modified QDs (605 nm emission) were conjugated to a bifunctional crosslinker BS3 to activate the surface of the QDs with highly labile succinimidyl groups. Activated QDs were purified over a NAP-5 column and subsequently conjugated with anti-GFP nanobodies (Fig. 1a schematic). QD-nanobody conjugate was characterized by gel electrophoresis and dot blot against purified GFP. When loaded in an agarose gel, the QD-nanobody conjugates migrate slightly slower than unconjugated or BS3 conjugated free QDs (Fig. 1b). When loaded to a denaturing PAGE gel and stained with Coomassie Brilliant Blue, QD-nanobody conjugates showed presence of both QDs and nanobodies confirming both are part of the same complex (Fig. S1†). Functionality of the complex was tested with GFP protein purified from HeLa cells and subsequently blotted on a nitrocellulose membrane. When compared to BSA as a control, the QD-nanobody complex resulted in strong signal from the GFP only dot blot, while no signal was detected against the BSA dot blot (Fig. 1c) confirming that nanobodies can detect GFP post conjugation with QDs. Specificity of the complex was further tested by dot blot with GFP and incubating the blot with QD-nanobody conjugates or free QDs. As expected, upon excitation with UV light, membranes that were incubated with free QDs only showed GFP fluorescence, while membrane incubated with QD-nanobody showed strong orange fluorescence corresponding to 605 nm QDs (Fig. 1d), confirming the QD-nanobody conjugate can specifically detect GFP molecules *in vitro*. To test the performance of our probe against GFP tagged membrane proteins, we expressed NrCAM-(Neuron-glial related Cell Adhesion Molecule), a cell adhesion protein. NrCAM belongs to L1-type cell adhesion molecules that mediate axogenesis and can also act as a receptor for F3/contactin to regulate axonal outgrowth.^26^ We transfected HeLa cells with NrCAM tagged with GFP in its extracellular domain and incubated the cells with nanobody coupled or free QDs respectively. QD-nanobody conjugates specifically labeled the cell surface in GFP-NrCAM transfected cells while untransfected cells did not show specific labeling (Fig. 1e). In contrast, QD-nanobody conjugates showed minimal binding towards cells expressing cytoplasmic GFP tagged AnkyrinG thus confirming specificity of QD-nanobody conjugates towards GFP on the membrane surface (Fig. S2†). Fig. 1f shows mobility of QDs on the surface of the cells and adhesion contacts. QD-nanobody conjugates were then used to follow the surface diffusion of GFP. This revealed that the QD tracks exhibit two kinds of motion (i) highly diffusive in nature and (ii) much more restricted and less mobile (Fig. 1g) characteristic of NrCAM molecules.^26^

**Fig. 2:**
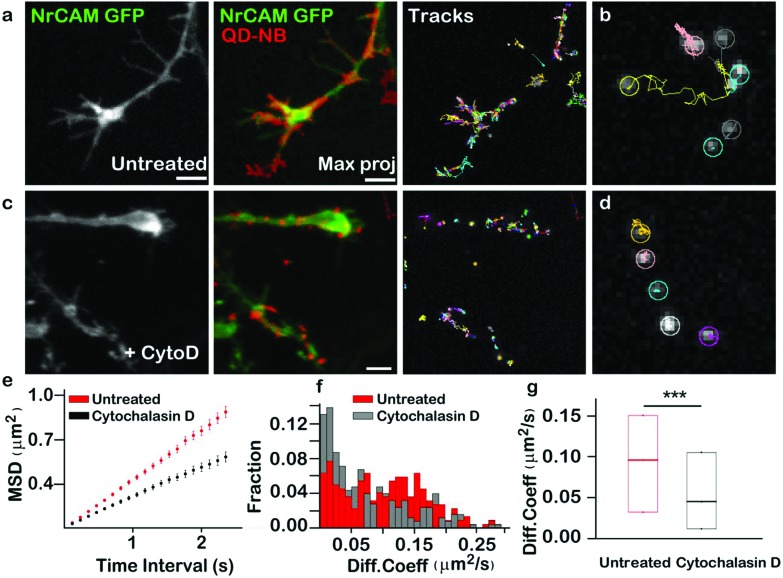
QD-nanobody conjugate revealed actin-dependent diffusion dynamics of NrCAM adhesion molecules at the growth cone of a developing axon of a hippocampal neuron. (a) A typical growth cone of a DIV 2 neuron was visualized with GFP-NrCAM (green). Maximum intensity projection of QD conjugate labelled NrCAM (red) was overlaid with GFP-NrCAM image to show dynamic NrCAM molecules covering entire growth cone within 25 s. Trajectories of QDs are shown in the right panel. (b) Zoomed region shows trajectories of individual QDs with free diffusion or more confined motion. (c) Altered morphology of the growth cone (green) upon incubation with Cytochalasin D. Maximum intensity projection of QD trajectories shows reduced mobility of QDs due to disruption of F-actin and their increased confinement to a local region instead of covering the entire growth cone (red). Images shown on right hand side represent trajectories of individual QDs. (d) Representative trajectories of QD conjugates before treatment and after treatment with Cytochalasin D. After depolymerization of actin, trajectories show more stationary behavior than untreated cells. (e) MSD plot of trajectories over 20 time points either in control cells or treated cells. (f) Distribution of diffusion coefficients measured from the MSD in two different conditions. Data used here are the same as shown in (c). (g) Diffusion coefficient of NrCAM molecule before and after treatment with Cytochalasin D. Median diffusion coefficient from ∼200 individual trajectories were shown. Three independent experiment, *n* = 25 cells for untreated, n = 38 cells for treated, *P*-value 8 × 10^–7^ (Mann–Whitney). QD-NB: QD-nanobody conjugate. Scale bar: 5 μm.

Next, we applied QD-nanobody labeling to the study of two different kinds of membrane protein dynamics (adhesion proteins at the growth cone and neurotransmitter receptors at synapses). Adhesion molecules are a class of membrane protein that maintain cell to cell contacts as well as cell migration with the help of cytoskeletal proteins. Although the interaction of L1 type of adhesion proteins and actin flow was previously reported using latex beads, anti-L1/CAM antibodies and laser tweezer methods, direct measurements of NrCAM diffusion at the growth cone using optical probes has thus far remained unexplored.^26^ To address this, we investigated the dynamics of NrCAM adhesion molecules during early stages of axonal growth by expressing GFP-NrCAM in dissociated rat hippocampal cultures and labeling with QD-nanobody conjugate. After 2 days *in vitro*, neurons expressing GFP-NrCAM were labeled with QD-nanobody conjugates and time-lapse images of the growth cones, where NrCAM is most dynamic, were acquired. Fig. 2a shows a typical example of an early stage (DIV 2) growth cone with QD labeled proteins diffusing throughout the growth cone, confirming their dynamic nature. Interestingly, QD-labeled NrCAM molecules showed two distinct types of trajectories. In the first type of trajectory, QDs were found to be restricted in a region for a long period of time, while the second type of trajectory showed a brief confinement followed by fast long-range movement (Fig. 2b). These types of NrCAM mobilities at growth cones are linked to local actin flow and stabilization of NrCAM molecules at adhesive contacts that advance the growth cone.^26^ It has been previously reported that reducing F-actin rearward flow by pharmacological treatment significantly alters diffusion of NrCAM molecules.^26^ To test this effect using our QD-nanobody labeling approach, we treated the cells with Cytochalasin D, which depolymerizes actin filaments, followed by labeling with QD-nanobody conjugates and imaged in a perfusion chamber with constant flow of imaging buffer containing cytochalasin D. Compared to untreated cells, depletion of actin filaments severely disrupted growth cone dynamics (Fig. 2c), leading to restricted QD diffusion. Furthermore, most of the NrCAM molecules showed stationary behavior consistent with earlier reports using TAG beads (Fig. 2d). In order to quantify this effect, MSD (Mean Square Displacement) curves for labeled NrCAM molecules in each condition were plotted and compared. In comparison to the untreated condition, NrCAM molecules in Cytochalasin D treated neurons exhibit decreased MSDs and sub-linear nature over elapsed time, indicative of increased confinement (Fig. 2e). This confinement is further highlighted with a histogram plot of all the observed QD diffusion coefficients (Fig. 2f), which shows that upon treatment with Cytochalasin D, there is an increase in QD-labelled NrCAM molecules exhibiting slow diffusion (<0.05 μm^2^ s^–1^) compared to untreated cells where more QD-labelled NrCAM molecules showing fast diffusion >0.1 μm^2^ s^–1^ could be detected. Upon quantification, NrCAM molecules in untreated conditions showed a median diffusion coefficient of 0.09 μm^2^ s^–1^ while in cytochalasin D treated cells NrCAM showed a significantly decreased diffusion coefficient of 0.045 μm^2^ s^–1^. These initial experiments revealed that the QD-nanobody conjugates can be applied to study lateral diffusion of membrane proteins with precision and reliability.

**Fig. 3:**
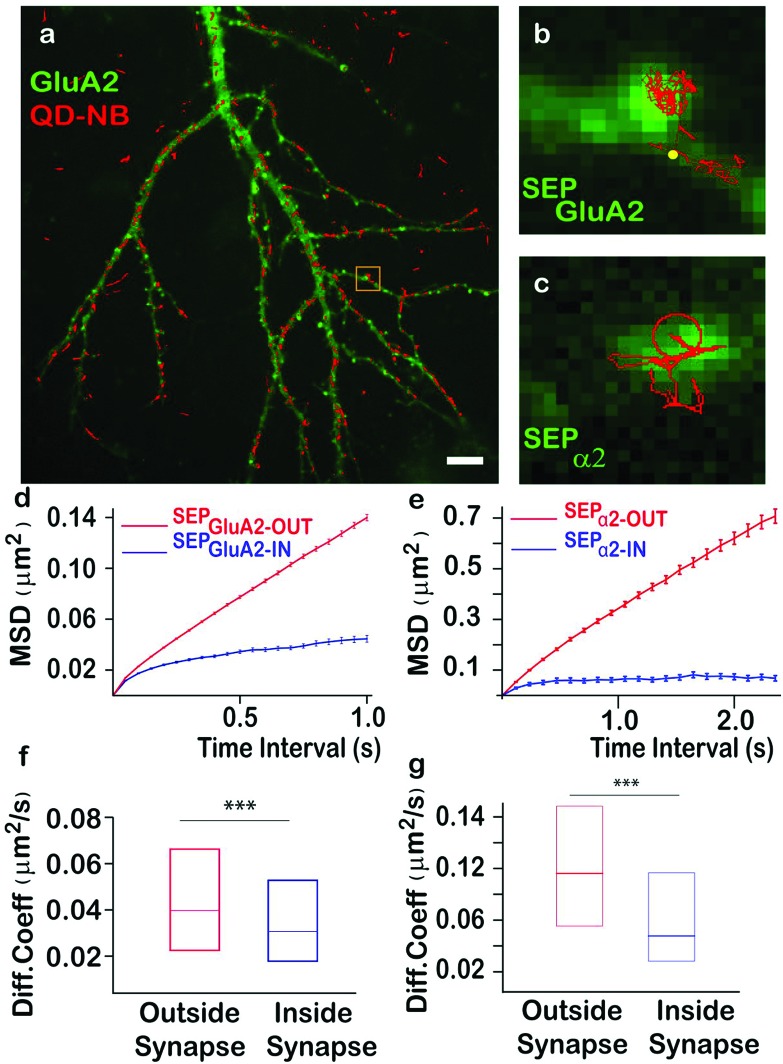
QD-nanobody conjugate revealed differential dynamics of AMPA receptors and GABA_A_ receptors at excitatory and inhibitory synapses, respectively. (a) QD-nanobody trajectories overlaid on rat hippocampal neurons expressing GluA2-SEP. (b, c) Representative trajectories of QD conjugates in neurons expressing GluA2-SEP at excitatory synapses (b) and in neurons expressing α2-SEP at inhibitory synapses (c). (d, e) MSD plot of QD trajectories present in extrasynaptic and synaptic regions in cells expressing GluA2-SEP and α2-SEP, respectively. Deviation from the linearity in all MSD curves present inside synapses confirmed more confinement of the receptors. (f, g) Diffusion coefficient of AMPA and GABA_A_ receptors outside the synaptic zone or inside synapses in cultured neurons showed a broad range. AMPA receptors showed much slower diffusion both outside and inside synapses (f) than corresponding GABA_A_ receptors (g). For AMPA receptors, 6041 extrasynaptic trajectories and 1090 synaptic trajectories are analyzed. *P* = 4.59 × 10^–18^. For GABA_A_ receptors, ∼440 extrasynaptic trajectories and ∼114 synaptic trajectories are analyzed. *P* = 2.63 × 10^–10^ Scale Bar: 5 μm.

**Fig. 4:**
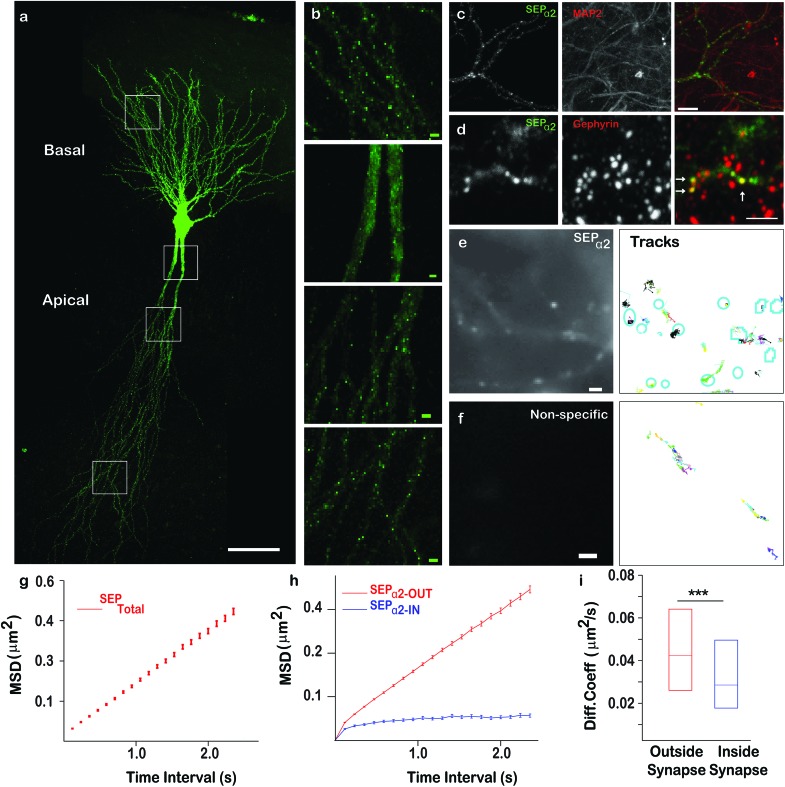
GABA_A_ receptor dynamics in *ex vivo* brain slices. (a) Confocal image of a pyramidal neuron in an organotypic brain slice biolistically transfected with α2-SEP. A representative neuron with bright α2-SEP foci at inhibitory synapses in apical and basal dendrites. (b) Enlarged view of four representative regions (white boxes in a) in basal and apical dendrites or close to soma. (c) Immunofluorescence of neurons transfected with α2-SEP (green) and co-labeled with the neuronal marker MAP-2 (red) showing their colocalization. (d) Immunostaining of transfected neurons co-labeled with the synaptic marker gephyrin (red) confirming α2-SEP localization at inhibitory postsynaptic domains. (e) Magnified view of the α2-SEP GABA_A_ receptor bound QD trajectories. (f) QD trajectories in a untransfected region was chosen as a control to show typical nonspecific labeling of QD-nanobody conjugate. (g) MSD plot of all QD trajectories – present on transfected neurons are shown while in (h) QD-nanobody conjugate trajectories present in extra synaptic and synaptic regions, shown respectively. (i) Diffusion coefficients (median ± 25–75% interquartile range (IQR)) of extrasynaptic and synaptic α2-SEP GABA_A_ receptors in organotypic brain slices. Median diffusion coefficient from ∼1951 individual trajectories for extra synaptic and ∼221 synaptic trajectories was shown. *P* = 0.009 (Mann–Whitney). Scale bar: (a) 50 μm, (b, d, e, f) 2 μm, (c) 5 μm.

Our results obtained from HeLa cells and cultured neurons highlighted that QD-nanobodies could be used for SPT of membrane proteins irrespective of cell types. QD-nanobody conjugates could improve accessibility to synaptic sites or to more complex diffusion environments such as intact tissues where accessibility and penetration have remained a major challenge for antibody conjugates. We therefore extended our study to the diffusion dynamics of neurotransmitter receptors into and out of synapses. α-Amino-3-hydroxy-5-methyl-4-isoxazolepropionic acid-type (AMPA) glutamate receptors in the mammalian CNS mediate the majority of excitatory synaptic transmission and their diffusion into and out of excitatory synaptic sites has been well-characterized in *in vitro* dissociated cultures.^5^,^27^ Synaptic inhibition mediated by γ-aminobutyric acid (GABA) also plays a critical role in regulating neuronal excitability^28^ and is similarly dependent on the number of GABA_A_ (GABA_A_) receptors present at inhibitory synaptic sites.^28^ As a prelude to diffusion studies in *ex vivo* slices, we tested the performance of the QD-nanobody conjugates in hippocampal neuronal cultures transfected with either the AMPA receptor GluA2 subunit or the GABA_A_ receptor α2 subunit bearing an N-terminal superecliptic phluorin (SEP) tag (a GFP variant that is recognized by the nanobody) at DIV 12 and imaged at DIV 14. As expected the QD-nanobody conjugate specifically labeled transfected neurons and single QD-labeled AMPA receptors (here after GluA2-SEP) could be seen to rapidly diffuse into and out of GluA2-SEP receptor clusters (which could be attributed as excitatory synapses) that were recognized as bright foci (Fig. 3a and b). Mean Square Displacements from single molecule trajectories of individual receptors revealed that the diffusion coefficients of synaptic or extra-synaptic AMPA receptors were significantly different with receptors inside synaptic regions diffusing significantly slower (curved MSD plots) than extra-synaptic ones (linear MSD plots) (Fig. 3d). In our hands, the median diffusion coefficient of extrasynaptic AMPA receptors was 0.039 μm^2^ s^–1^ while synaptic receptors showed significantly decreased mobility with diffusion coefficients of 0.030 μm^2^ s^–1^ (Fig. 3e). These results confirmed that the QD-nanobody conjugate labelled receptors can access synaptic densities similar to conventional antibody labeling approaches with QDs.^21^,22d We similarly tested dynamics of GABA_A_ receptors in dissociated cultures using QD-nanobodies. When neurons were transfected with the α2 subunit of the GABA_A_ receptor fused with a SEP tag (here after α2-SEP) and subsequently labeled with QD-nanobody, it showed specific labeling of QD on transfected neurons similar to GluA2 transfected neurons (Fig. 3c and S3†). Upon quantification, we observed that the diffusion coefficient of synaptic GABA_A_ receptors was significantly slower than the extra-synaptic receptors (Fig. 3f and g). Importantly, the QD conjugated nanobodies detected distinctive diffusion coefficients for the receptor subunits inside and outside synapses. This confirmed QD-nanobodies can be used to track most types of membrane proteins/receptors available as chimeric GFP fusions, regardless of their nature and locations on dendrites and spines. Mechanistic information about diffusion of neurotransmitter receptors has been primarily obtained from experiments in *in vitro* dissociated cultures, while currently far less information is available regarding their dynamics in more intact tissues. *Ex vivo* brain slices are a good model system that more closely resembles *in vivo* neuronal networks, preserving endogenous connections between neurons and neuron-glial interactions. Although synaptic diffusion of GABA_A_ receptors has been reported in dissociated cultures, their mobility and distribution in intact tissues remain unexplored. Due to their smaller size and great brightness, the QD-nanobody conjugates are well suited to explore dynamics of receptors in molecularly crowded environments, such as within organotypic brain slice, where each neuron is compactly arranged in a 3D environment.^29^ Rat organotypic brain slices were cultured for 7 days and then subjected to biolistic transfection with α2-SEP. Slices were further cultured for 7 days and then either fixed for immunostaining or labeled with QD-nanobody conjugates to explore receptor dynamics. Fig. 4a shows a pyramidal neuron in the CA1 hippocampal region transfected with α2-SEP which forms bright clusters along apical and basal dendrites. Immunostaining with MAP-2 and gephyrin respectively confirmed that these clusters are located at inhibitory post synaptic sites in dendrites (Fig. 4b–d). To study receptor dynamics in *ex vivo* slices, transfected slices were further labeled with QD-nanobody conjugates. We mainly observed highly specific labeling of QDs enriched in the transfected neuron with minimal nonspecific labeling of adjacent untransfected cells (Fig. 4e and S4†). We noticed two kinds of trajectories, (i) slightly less mobile QD-nanobodies concentrated on transfected cells (identified from SEP fluorescence) and (ii) fast moving QDs not specific to any kind of cells which could be attributed to free QDs moving through the extracellular space (Fig. 4e, f and S5†).29*a* We only focused on the first type of trajectories and discarded those trajectories that were not localized on transfected cells. Overall analysis of QD trajectories on transfected neurons revealed two types of movement, one where QDs are confined within a small region (often colocalized with SEP clusters) and another type where QDs are freely diffusing along a dendrite with occasional confinement (Fig. 4e). This observation enabled us to delineate the QD trajectories that were either extrasynaptic (trajectories of QDs with high diffusion coefficients outside the synapse) or synaptic (confined QD trajectories inside bright SEP clusters). Fig. 4 g represents the MSD of GABA_A_ receptors confirming their diffusive nature *ex vivo*. Interestingly, when extrasynaptic trajectories were separated from the trajectories inside synaptic zones, we observed more confinement of synaptic receptors (median diffusion coefficients ∼0.042 μm^2^ s^–1^ for extrasynaptic receptors and ∼0.028 μm^2^ s^–1^ for synaptic receptors) (Fig. 4h and i). Our result showed that similar to cultured neurons, synaptic receptors in intact brain slices are less mobile than extrasynaptic GABA_A_ receptors.

**Fig. 5:**
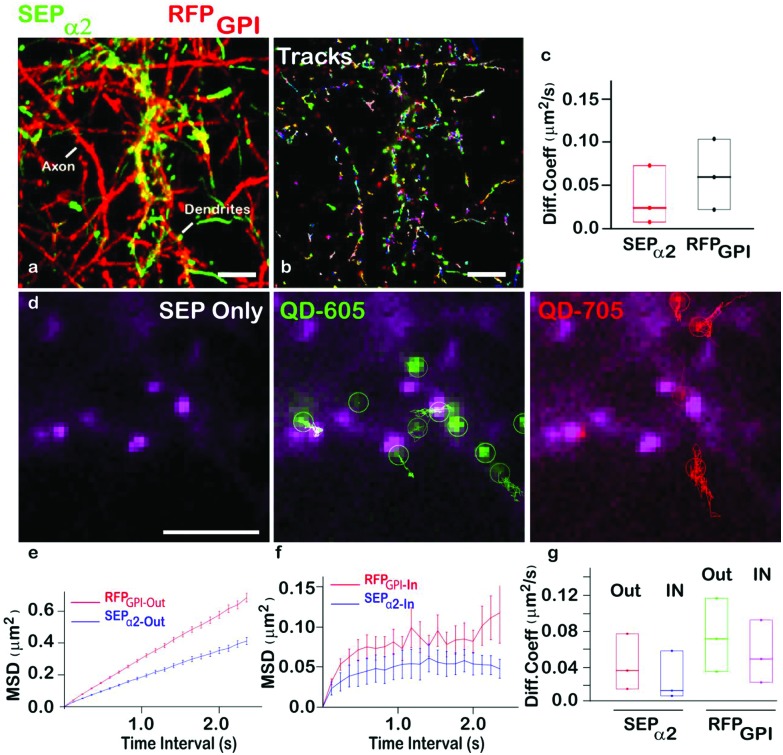
Multiplexed imaging of GABA_A_ receptors and GPI anchored proteins. (a) Simultaneous labeling of GABA_A_ receptors with QD^605^ and GPI anchored proteins with QD^705^ in dendrites and axons. Maximum intensity projections of QD^605^ (green) and QD^705^ (red) over time revealed area explored by both proteins. (b) Individual trajectories of QD^605^ and QD^705^ in a given field of view is shown. (c) Diffusion coefficient of GABA_A_ receptors (green) and GPI anchored proteins (red) quantified from the same neuron reveal different mobilities of the two proteins. (d) Detection of GABA_A_ receptors (green) and GPI anchored proteins (red) in the same synapse (magenta, white arrow). (e, f) Mean Square Displacements of GABA_A_ receptors (blue) and GPI anchored proteins (red) in extrasynaptic (e) and synaptic regions (f) showed that GABA_A_ receptors are significantly more confined in comparison to GPI anchored proteins. (g) Diffusion coefficient of GABA_A_ receptors and GPI anchored proteins in extra synaptic and synaptic regions. Scale bar: (a) 10 μm, (d) 5 μm.

To further explore our QD-nanobody approach, we conjugated 705 nm QD conjugated to RFP nanobody in order to perform multiplex imaging of two membrane proteins in the same dendritic shaft and synapses. Rat hippocampal neurons were transfected with α2-SEP (to label synaptic sites) and GPI-RFP (to label whole neuronal membranes) and label them with QD-nanobody conjugates. Neurons were imaged in a widefield microscope that contained an image splitter in front of the camera to separate 605 nm and 705 nm emission wavelengths. This enabled simultaneous imaging of GABA_A_ receptors and GPI anchored proteins in the same synaptic regions. Neurons expressing both markers showed labelling of QD^605^ and QD^705^ nanoprobes (Fig. 5a and b) against SEP tagged GABA_A_ receptors and RFP tagged GPI anchored proteins, respectively. MSDs from individual trajectories of QD-labeled GABA_A_ receptors or GPI anchored proteins on inhibitory synapses (marked by α2-SEP clusters) revealed that diffusion coefficient of synaptic or extra-synaptic GABA_A_ receptors or GPI anchored proteins are different (Fig. 5c and e), with GPI anchored proteins found to be fast diffusing29*a* while the mobility of GABA_A_ receptors was significantly slower. This trend is maintained when we delineate synaptic trajectories and extrasynaptic trajectories (Fig. 5d and f). Our results also revealed that diffusion of synaptic GABA_A_ receptors (∼0.015 μm^2^ s^–1^) are significantly slower than synaptic GPI anchored proteins (∼0.042 μm^2^ s^–1^) (Fig. 5g). This is not surprising given that synaptic GABA_A_ receptors often interact with scaffolding proteins (*e.g.* gephyrin) which in turn stabilizes them within synapses. Our data demonstrate that QD-nanobody conjugate can precisely differentiate the dynamics between two molecules within the same compartment.

## Conclusions

This report demonstrates the application of QD conjugated nanobodies for single particle tracking of tagged membrane proteins in single cells and intact tissue. QD-nanobody or recombinant antibody (V_H_H only) conjugates *e.g.* anti-EGFR conjugated to QDs have been used to track monomeric and dimeric EGF receptors.30*a* On the other hand, four nanobody conjugated QDs have been used to detect carcinoembryonic antigen (CEA) while single domain antibody against Her2 has been used to detect breast and lung cancer cells.30b,c Recent development of small QD-Nanobody conjugate as well as super-resolution techniques *e.g.* uPAINT (Universal Point Accumulation in Nanoscale Topography)22e,f revealed that these smaller probes can access the synapse better than conventional antibody conjugated probes.22b,c,d,g Although these reports explored several aspects of protein detection, diffusion and imaging, none of these studies fully utilized their potential for SPT of multiple membrane proteins from same compartments, from same cells or reported neurotransmitter receptor dynamics *in situ* in brain slices.

We specifically chose to study three different aspects of membrane protein dynamics. First, we demonstrated actin mediated diffusion of NrCAM adhesion molecules at growth cones. During early stages of neuronal growth, axons grow at a fast rate dependent on actin dynamics. Our result with QD-nanobody conjugates revealed that during early axon generation retrograde F-actin flow determines the dynamics of the NrCAM adhesion molecule.

Second application of QD-nanobody conjugates revealed neurotransmitter receptor dynamics at synapses in cultured neurons as well as intact brain tissues. Synapses have a small confined volume containing a high density of neurotransmitter receptors. AMPA receptor diffusion dynamics inside synapses obtained with our QD-nanobody conjugates remain comparable with published literature which highlighted the performance of QD-nanobody conjugates at the synaptic cleft.^22^ Moreover, superior photostability of QD-nanobody conjugate compared to organic dyes provides an extra advantage for imaging over longer periods of time.^15^,^21^,^22^,^30^–^37^ To further extend the application of QD-nanobody conjugate, GABA_A_ receptor dynamics at inhibitory synapses in cultured neurons and organotypic brain slices were investigated. In cultured neurons, GABA_A_ receptors at synapses showed diffusion coefficient of 0.047 μm^2^ s^–1^ which is in good agreement with earlier studies.^38^ When rat brain slices were transfected with α2-SEP and labeled with QD-nanobody conjugate, it specifically marked GABA_A_ receptors in α2-SEP-containing neurons with minimal non-specificity. In combination with live cell imaging, this QD-nanobody conjugate revealed the highly dynamic nature of GABA_A_ receptors in the extra synaptic region while exhibiting a more confined motion inside inhibitory synapses in *ex vivo* brain slices as well as dissociated cultures *in vitro*. We observed slower diffusion of GABA_A_ receptors (both synaptic and extra synaptic receptors) in brain slices compared to those observed in dissociated cultures (synaptic diffusion coefficient ∼0.028 μm^2^ s^–1^*ex vivo* compared to 0.047 μm^2^ s^–1^ obtained in cultures). This data supports the possibility that the diffusion environment of synapses in brain slices is more restricted than that of synapses formed in dissociated cultures.

Third application showed simultaneous imaging of two different membrane proteins in the same cell as well as in similar compartments. Due to small size, high specificity and easy conjugation strategy, we labeled multiple QDs to different antibodies to perform multiplexed imaging. Our data showed that QD-nanobody conjugates could be successfully used to label two membrane proteins in the same neuron without major cross-reactivity between them. Orthogonal imaging of two proteins revealed contrasting diffusion coefficients in the same dendritic shaft. We found that GPI anchored proteins diffuse almost 3 times faster than GABA_A_ receptors. When compared to their diffusion behaviour in more molecularly crowded environments such as synapses, we find that GABA_A_ receptors are more confined inside synapses than GPI anchored proteins. This extra stabilization of synaptic GABA_A_ receptors could be contributed by molecular interaction of the receptors with scaffolding proteins, such as Git1, Gephyrin, or LHFPL4.25c,^39^,^40^

We believe that QD-nanobodies are well suited to study axonal and dendritic trafficking *in vitro* or *ex vivo* during neuronal development. Application of QD-nanobodies is not limited to the study of neurotransmitter receptors or adhesion molecules; it could also be used to track other membrane proteins bearing an extracellular GFP (or RFP) tag. One limitation of tracking with QD-nanobodies to fluorescent proteins is the requirement of a tagged target protein, which currently requires its transfection as in our current study. However recent advances in CRISPR-Cas9 techniques that allow GFP tagging of endogenous proteins including membrane proteins, will greatly open up the applicability of QD-nanobody probes to tracking endogenous proteins in intact tissues.^41^ Moreover, its small size and high affinity makes it ideal to study protein dynamics at membranes or in intact tissues, where accessibility of bigger probes may be restricted, such as protein organization inside clathrin coated pits or endocytosis of synaptic vesicles and their trafficking inside dendrites or axons. With its relatively easy conjugation with smaller antibodies, high specificity and photostability, QD-nanobodies offers an excellent platform to simultaneously investigate multiple complex biological phenomena, like receptor dynamics during synaptic signalling *in vivo*.

## Conflicts of interest

There are no conflicts to declare.

## Supplementary Material

Supplementary movie

Supplementary movie

Supplementary movie

Supplementary movie

Supplementary movie

Supplementary information
