# Supplementary material for: Quantum dot conjugated nanobodies for multiplex imaging of protein dynamics at synapses
Source: Nanoscale. 2018 May 17;10(21):10241–9. doi: 10.1039/c7nr09130c (PMC5977936; doi:10.1039/c7nr09130c)
Supplement: Supplementary file 6 [file NR-010-C7NR09130C-s006.pdf]

Electronic Supplementary Material (ESI) for Nanoscale.
This journal is © The Royal Society of Chemistry 2018

Supporting information for

Quantum Dot conjugated nanobodies for multiplex imaging of protein dynamics at synapses

Materials and methods

Quantum Dot functionalization with nanobody

Qdot 605 ITK™ Amino (PEG) Quantum Dots were purchased from Life Technologies. 37 µl of QD605 was mixed with 13 µl 1M Borate buffer, pH 8, 0.5 mg Bis[sulfosuccinimidyl] suberate (BS3) (Sigma Aldrich) and incubated for 30 min at RT with mild vortex. This mixture then loaded onto a Nap-5 column (Life technologies) and QD-BS3 conjugate was eluted with 1×PBS pH 7.4. First 500 µl coloured solution was collected and concentrated to 50 µl using a 50 kDa molecular weight cut-off filter (Life Technologies). 100 µl anti-GFP nanotrap (nanobody hence after) (ChromoTek GmbH) was added to the QD conjugate and incubated for 2h at RT with mild vortex. QD and nanobody mixture then diluted to 400 µl and loaded on to a 50 kDa molecular weight cut-off filter and centrifuged at 4000×g for 8 min. Flow through was discarded and supernatant was diluted to 400 µl and centrifuged again. This step was repeated 5-6 times and then the supernatant was concentrated to 50 µl. Finally unreacted BS3 sites on QD-nanobody conjugates was quenched by adding 2 µl of 1M Tris buffer pH 7.4.

Characterization of QD-nanobody conjugates *in vitro*

QD conjugates, free QD and QD-BS3 were analysed using 1% Agarose-TBE gel. Gel was ran for 2-3 h at 100 V and visualized under UV in a gel documentation system. These conjugates were further analysed with 12% SDS page and stained with coomassie brilliant blue to visualize protein conjugates and free nanobodies. GFP was purified from HeLa cells by transfecting them with CAG GFP followed by affinity capture using GFP trap agarose beads (ChromoTek GmbH). Affinity captured GFP was eluted from bead by addition of 50 µl 1M glycine pH 2.5 and then quenching with 1M Tris pH 7.4. 5 µl of GFP and BSA (1 mg/ml) was blotted in a nitrocellulose membrane and allowed to dry at RT for 30 min. The membrane was blocked with PBST (PBS+0.05% Tween-20) containing 10% Horse serum (Life Technologies) and 1% BSA. The blot was incubated with 1 in 10000 dilution of QD-nanobody conjugates overnight at 4ºC. The membrane was washed 3 times in PBST and developed in a UV illuminated gel documentation system. Dot blot with QD-nanobody conjugates and free QD was performed similarly with minor modifications. For labelling, 1 in 5000 dilution of QD-nanobody conjugates and 1 in 2000 dilution of free QD was used.

Cell culture, transfection, labeling and staining

HeLa cells were cultured in DMEM medium (Gibco) supplemented with streptomycin (100 µg/ml), penicillin (100 U/ml), and 10% fetal bovine serum on 13 mm glass coverslips in a 6 cm cell culture dishes. Cells were transfected with ~5 µg of GFP-NrCAM or GFP-AnkG

using nucleofection (Amaxa, Lonza AG). After 24h post transfection, cells were labelled with QD-nanobody conjugates (1 in 3000 dilution) for 5 min at RT in PBS containing 10% Horse serum. Coverslips were washed 3 times by dipping in PBS and imaged in a widefield set up based on Olympus BX51WI microscope with a 60× Olympus objective coupled to an EM-CCD camera (Andor) and a perfusion chamber as described in.[1] Images were acquired at 8.4 Hz for 20-30s and analysed in ImageJ (NIH).

Rat hippocampal cultures were performed as described earlier and transfected on the day of dissection using nucleofection with 10 µg plasmid DNA either of GFP-NrCAM (A gift from Dargent Lab) or with 5µg of α2-SEP (Gift from S. Moss) according to manufacturer's protocol.[1] 2 days post transfection with GFP-NrCAM, neurons were labelled with 1 in 1000 dilution of QD-nanobody conjugates for 5-7 minutes and washed 2 times with PBS and imaged as described above using 405 illuminations of QDs.[1] Growth cones were imaged after identifying axon initial segment that generally have higher GFP expression. For depletion of F-Actin network, neurons were treated with 39 µM Cytochalasin-D (Sigma) in their original culture media for 30 min at 37ºC and then labelled with QD-nanobody conjugates containing 39 µM Cytochalasin-D at RT for 5 min.[2] Cells were washed twice in PBS and imaged with continuous perfusion of imaging media containing 1 µM Cytochalasin D. α2-SEP expressing neurons were imaged 14 day post nucleofection after labelling with QDs (1 in 3000 dilution) as described above.

Organotypic slice culture, transfection and labeling

Organotypic hippocampal slice cultures were made following the Stoppini interface method (2), where by cultured slices are maintained on membrane insert.[3] Briefly, P7-10 Sprague-Dawley rats were sacrificed in accordance with schedule 1 Home Office protocol. The brain was removed and placed in a petri dish containing ice-cold slicing media (EBSS + 25mM HEPES). 300 µm sagittal slices were made on a vibratome (Leica) following which the hippocampus was dissected and placed on the membrane. Slices were cultured in an incubator containing 5% CO_2 for 7 days at 37ºC in a 6 well plate containing 1.6 ml of culture media (72% MEM (Gibco), 25% Horse Serum, 0.64% Glucose (Sigma), 100 U/ml Penicillin, 100 µg/ml Streptomycin, 6 U/ml Nystatin (Sigma)). Slices were fed with fresh culture media every 3-5 days and transfected with a hand-held Helios gene gun (Bio-Rad) 7 days post dissection. 50 µg DNA was coupled to gold microcarriers (1.6 µm diameter) by precipitation with calcium chloride according to the previous literature[4,5]. Finally, the gold coated cartridges were inserted inside the gene gun and shot onto the slices with helium pulse. Slices were further cultured for 7 days as described above and labelled with QDs. Slices with membranes were removed from the wells and 1 in 1000 dilution of QD-nanobody conjugates diluted in PBS+10% horse serum+1% BSA was added to the slices and incubated for 5-8 minutes unless specified, at 37ºC. Slices were washed 2 times in PBS and imaged as described earlier.

Immunofluorescence and imaging

Slices were fixed with 4% paraformaldehyde containing 4% Sucrose for 10 minutes in PBS and washed 5 times with PBS. Slices were transferred in a well of 24 well plate and

permeabilized with 0.2% TritronX-100 in PBS containing 10% horse serum and 1% BSA for 2 h at RT. Slices were then labelled with anti-GFP Rat IgG (Nacalai Tesque, 1 in 2000), anti-Gephyrin Mouse IgG (Syanptic System, 1 in 250) and anti-MAP2 guinea pig IgG (Synaptic Systems1 in 500) at 4ºC overnight in a eppendorf thermomixer with gentle shaking. Slices were washed 3 times with large volume of PBS with shaking and labelled with secondary antibodies for 2 h at RT. 1 in 500 dilutions of Alexa-488 conjugated anti-Rat; Alexa-555 conjugated anti-Mouse and Alexa-647 conjugated anti-Guniea pig (Obtained from Molecular probes, Life Technologies) were used to visualize various molecular markers. Slices were mounted in Vectashield H-1400 (Vector labs) and imaged in a zeiss LSM 700 confocal microscope with a plan-apochromat 63× oil-immersion lens with 1.4 numerical aperture. Z stacks were acquired with 1 μm step size and then projected using maximum intensity projection in ImageJ.

QD Image Analysis

QD Detection and Tracking

Quantitative analysis of QD recordings can be separated into two discrete steps: detection and tracking. Accurate QD detection was achieved using an image segmentation algorithm called Basic Image Features (BIFs) which had previously been employed in a SPT study.[1] Briefly, a set of 2-D Gaussian filters is applied to each frame so that each pixel is classified as one of seven different basic image features (BIFs).[6,7] Pixels that possess high local rotational symmetry, corresponding to local fluorescence maxima, are classified and grouped accordingly. As QDs appear as local diffraction-limited bright blurs, these BIFs pixel clusters are considered QDs. Given a set of detected QDs for each frame, trajectories were formed using tracking software written in Mathematica (Wolfram Research).[1] The algorithm works by minimising the Euclidean Distance between QD tracks across consecutive frames. It can account for QD blinking by allowing QD tracks to not link to any specific QD in one frame, only to link to a re-emergent QD in a subsequent frame. The combinatorial complexity of the algorithm is assisted by the user-specified maximum distance a QD is capable of moving across successive frames. Furthermore, this step limits the search for linked QDs to a bounded distance (no. frames since detection × max distance). This maximum interframe distance was generally set at 2 pixels (0.55μm) per timestep (120 ms). Trajectories were required to consist of at least 50 points to be included in the analysis. Mean square displacement (MSD) versus time (t) was calculated for each QD track using the formula:

$$\mathrm{MSD}(n*dt) = (N-n)^{-1}\sum_{t=1}^{N-n}((x_{t+n}-x_t)^2+(y_{t+n}-y_t)^2))$$

where xi & yi are the Cartesian coordinates of the QD at frame i, dt is the timestep between frames, n is number of timesteps and N is total number of frames in the movie. Note that MSD (0) = 0. Instantaneous diffusion coefficients (D) were then estimated by fitting a line through the origin to the first five points of the MSD curve and using the 2D diffusion law (MSD = 4 D×t). QDs exhibiting diffusion score lower than 5×10^{-3} were considered immobile and removed from the subsequent analysis.[6-8] Similarly, trajectories that produce D > 1 were removed from the analysis. While the QD diffusion score was estimated by linear fit of the

first five points of the MSD, in some cases the MSD was plotted as a function of time (MSDt) for a population of QDs. In these instances, the MSD was calculated for each QD at each timestep for at least 20 points. Then, the values at each timestep were subsequently averaged to produce a single curve. The standard error around each averaged timestep was plotted. Some QD movies were acquired alongside a single image illustrating the position of synaptic puncta. These images were taken immediately before the QD movie. Synapse position was calculated from these images using the BIFs algorithm in a similar manner to the QD sub-pixel localisation. QD position was overlaid on synapse positions to determine whether trajectories were synaptic or extra-synaptic. A trajectory was considered synaptic if the distance between the two centroids (QD and synapse) was less than 2 pixels.[1] In these instances, single trajectories can return both an inside and outside diffusion score. Identical diffusion thresholds were applied to each set, as before ($0.5 \times 10^{-3} < D < 1$). Representative QD tracks were created in ImageJ using Mosaic particle tracker 2D/3D plugin.[9,10]

We examine drift by following methods, z project all the movies to assess if significant x-y drift is present in the movies. Any drift > 2.5 μm (i.e ~10 pixel) are discarded. Any drift smaller than that are corrected as described elsewhere using StackReg macro (Rigid body or translation algorithm) embedded in imageJ.[1]

SI figure 1

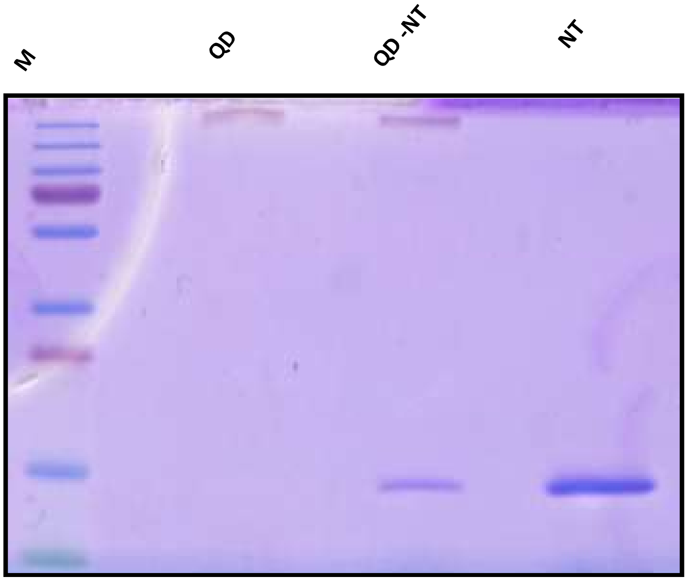

Figure S1: SDS page analysis of QD-nanobody conjugates. 12% SDS page was performed to confirm presence of Nanobody in QD-nanobody conjugates. M: PageRuler Plus Prestained Protein Ladder (Thermo Scientific); QD: Free Quantum dot; QD-NT: QD-nanobody conjugate; NT: Free nanobody. Gel was run at 100 V and stained with Coomassie Brilliant Blue R250.

SI Figure 2

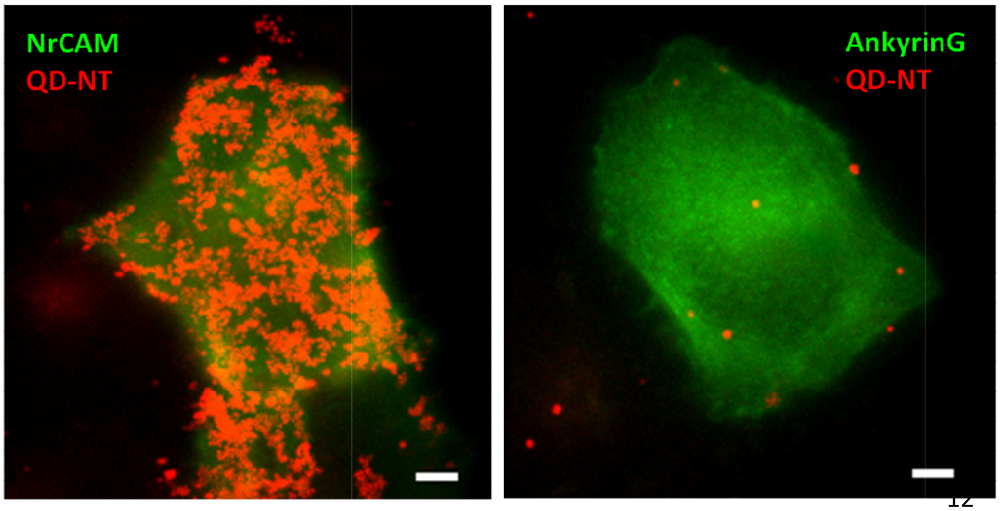

Figure S2: Specificity of QD-nanobody conjugates towards GFP. HeLa cells were transfected with either GFP-NrCAM or GFP-AnkyrinG as described in the methods section. 24h post transfections, cells were labelled with a 1 in 1000 dilution of QD-nanobody conjugates for 5 min and then washed 3 times in PBS and imaged with a Zeiss inverted microscope with 63× Plan-apochromat objective having photometrics evolve CCD camera under constant perfusion. Images were acquired at a rate of 1fps and projected using maximum intensity projection method in ImageJ. It can be seen that when GFP is present at the cell membrane (GFP-NrCAM), QD-nanobody conjugates recognizes it, while in ankyrinG expressing cells where GFP is expressed in the cytoplasm, no labelling is observed by the probe. Scale bar: 5 μm

SI Figure 3

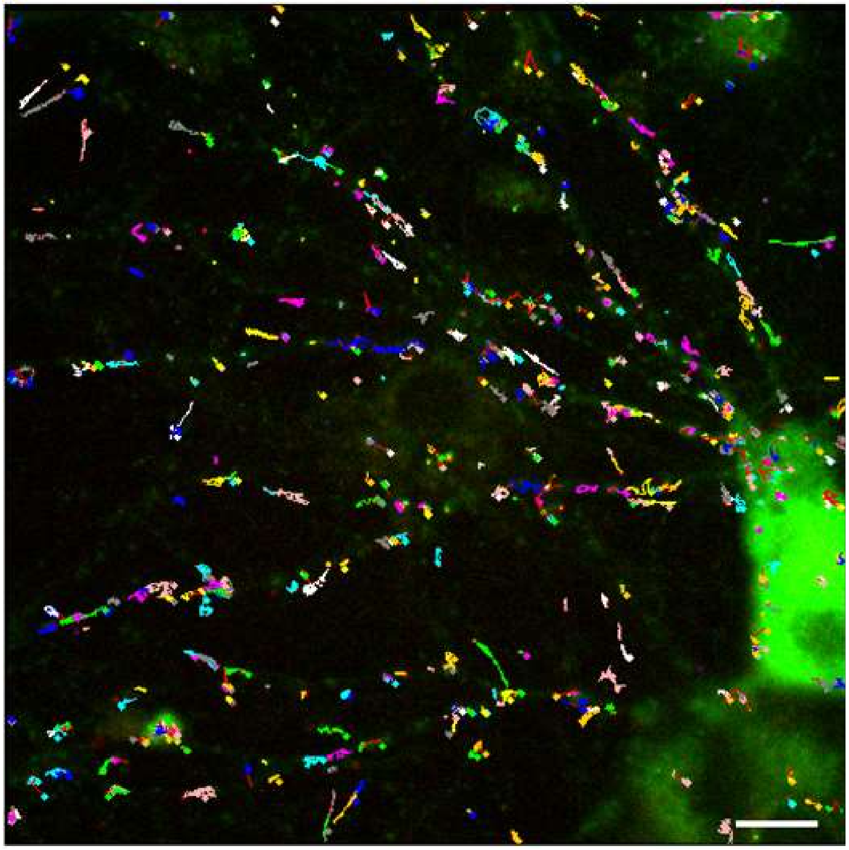

Figure S3: QD-nanobody conjugates trajectories overlaid on rat hippocampal neurons from post-natal day 0 rat cultures expressing $GABA_A$-SEP (α2-SEP). Transfection of α2-SEP was performed on the day of dissection using nucleofection as described in the methods section. After 12-14 days of transfection, neurons were labelled with QD-nanobody conjugates for 5 min at RT and imaged in an Olympus microscope with 60× objective coupled to an Andor CCD camera under continuous perfusion. Imaging was performed at 8.4 Hz and representative tracks were created and overlaid using a Mosaic plugin available in Image J. Scale bar: 10 µm.

SI Figure 4

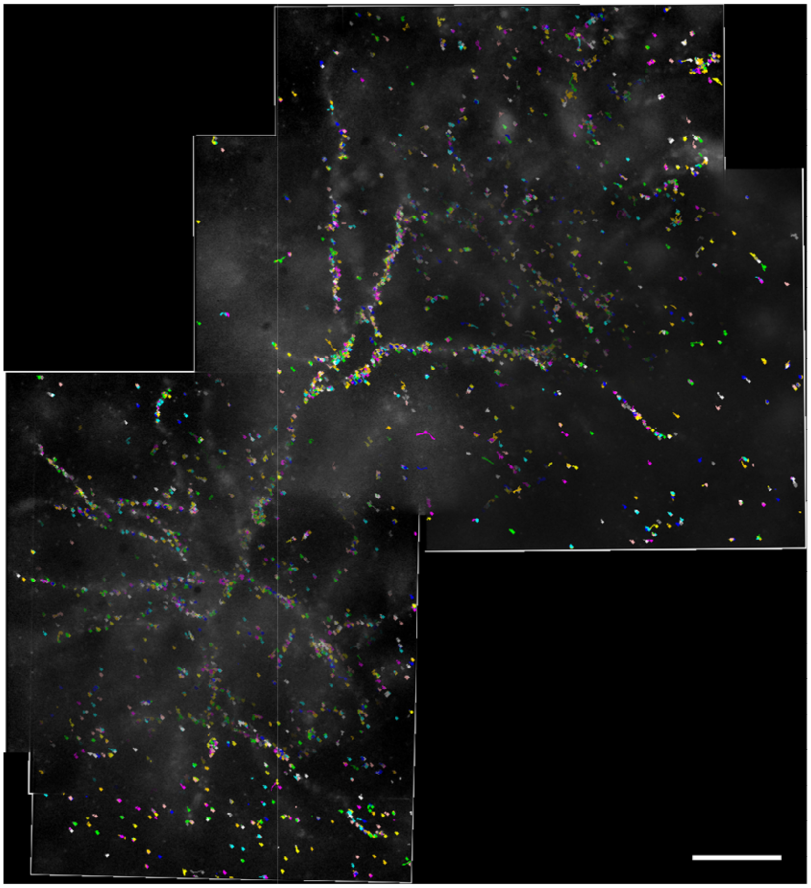

Figure S4: QD-nanobody conjugate trajectories in rat brain slices after longer exposure with QDs. Organotypic slices were transfected with α2-SEP using biolistic method and imaged 7 days post transfection. Slices were labelled with QD-nanobody conjugate for 30 min prior to imaging at 37ºC, washed 3 times and placed in a perfusion chamber attached to a wide field microscope. Transfected neurons were identified using GFP filters and then QD images were acquired at 8.4 Hz. Tracks were created in imageJ, overlaid with the GFP image and stitched using MosaicJ plugin available in ImageJ.[10] Note: Due to longer labelling protocol, mobile QDs not only labelled complete neuron but also increases nonspecificity which is reported in earlier literature.[4] Scale bar: 30 µm.

SI Figure 5

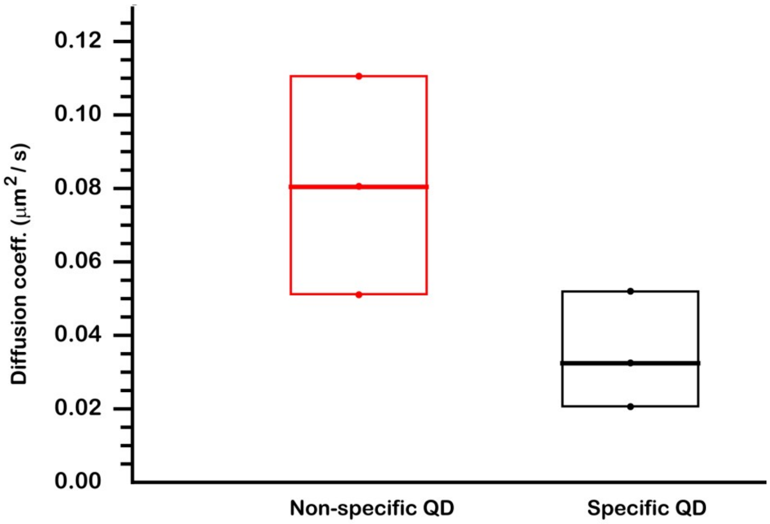

Figure S5: Dynamics of nonspecific QDs diffusing through extracellular space compared to specific QD-nanobody conjugate diffusing in dendrites. After transfection and labelling with QDs, nonspecific QDs (QD trajectories not present on transfected neuron) and specific QDs (QD trajectories present on dendrites) were identified and analysed as described earlier.

SI Video 1

GFP-NrCAM dynamics in untreated cells labelled with QD-nanobody conjugate.

SI Video 2

GFP-NrCAM dynamics in cells treated with Cytochalasin D followed by labelling with QD-nanobody conjugate.

SI Video 3

$GABA_A$ receptor dynamics in *ex vivo* slices labelled with QD-nanobody conjugate.

SI Video 4

A representative track of a $GABA_A$ receptor in *ex vivo* slices labelled with QD-nanobody conjugate showing confinement inside synapse.

SI Video 5

Simultaneous imaging of GABAA receptors (green) and GPI anchored proteins (red) at dendritic shaft and synapses (violet).

SI References:

(1) Muir, J.; Arancibia-Carcamo, I. L.; MacAskill, A. F.; Smith, K. R.; Griffin, L. D.; Kittler, J. T. *Proc. Natl. Acad. Sci. U.S.A.* **2010**, *107* (38), 16679–16684.

(2) Boevink, P.; Oparka, K.; Cruz, S. S.; Martin, B.; Betteridge, A.; Hawes, C. *The Plant Journal* **1998**, *15* (3), 441–447.

(3) Stoppini, L.; Buchs, P. A.; Muller, D. *J. Neurosci. Methods* **1991**, *37* (2), 173–182

(4) Biermann, B.; Sokoll, S.; Klueva, J.; Missler, M.; Wiegert, J. S.; Sibarita, J.-B.; Heine, M. *Nat Commun* **2014**, *5*, 3024.

(5) Woods, G.; Zito, K. *J Vis Exp* **2008**, No. 12.

(6) Griffin, L. D. *Journal of Mathematical Imaging and Vision* **2009**, *34* (3), 259–269.

(7) Griffin, L. D.; Lillholm, M. *IEEE Transactions on Pattern Analysis and Machine Intelligence* **2010**, *32* (6), 1072–1083.

(8) Papouin, T.; Ladépêche, L.; Ruel, J.; Sacchi, S.; Labasque, M.; Hanini, M.; Groc, L.; Pollegioni, L.; Mothet, J.-P.; Oliet, S. H. R. *Cell* **2012**, *150* (3), 633–646.

(9) Sbalzarini, I. F.; Koumoutsakos, P. *J. Struct. Biol.* **2005**, *151* (2), 182–195.

(10) Thévenaz, P.; Unser, M. *Microsc. Res. Tech.* **2007**, *70* (2), 135–146.
